# Characterization of the Highly Prevalent Regulatory CD24^hi^CD38^hi^ B-Cell Population in Human Cord Blood

**DOI:** 10.3389/fimmu.2017.00201

**Published:** 2017-03-07

**Authors:** Ana Esteve-Solé, Irene Teixidó, Angela Deyà-Martínez, Jordi Yagüe, Ana M. Plaza-Martín, Manel Juan, Laia Alsina

**Affiliations:** ^1^Allergy and Clinical Immunology Department, Hospital Sant Joan de Déu, Institut de Recerca Pediàtrica Hospital Sant Joan de Déu, Esplugues de Llobregat, Spain; ^2^Functional Unit of Clinical Immunology, Sant Joan de Déu-Hospital Clinic, Barcelona, Spain; ^3^Materno-Fetal Medicine Department Hospital Clínic de Barcelona (HCB), Barcelona, Spain; ^4^Immunology Service, Biomedic Diagnostic Center, HCB Universitat de Barcelona, IDIBAPS, Barcelona, Spain

**Keywords:** regulatory B-cells, newborn, tolerance, B-lymphocytes, umbilical cord

## Abstract

The newborn’s immune system must transition from a sterile haploidentical uterus to the world full of antigens. Regulatory B-cells (Breg; broadly defined as CD19^+^CD24^hi^CD38^hi^) are tolerance promoters in the adult immune system. They can inhibit IFN-γ and IL-17 production by T-cells and are essential in different conditions, including pregnancy. Breg have still not been well characterized in umbilical cord blood, where we hypothesize that they are pivotal in the achievement of tolerance. We studied CD19^+^CD24^hi^CD38^hi^ Breg in healthy umbilical cord blood (hUCB) compared to healthy peripheral adult blood (hAPB). Total numbers of Breg were increased in hUCB compared to hAPB (34.39 vs. 9.49%; *p* = 0.0002), especially in the marginal zone-like B-cell subset, in which the most marked difference could be observed between hUCB and hAPB (60.80 vs. 4.94%; *p* = 0.1). CD24^hi^CD38^hi^ subset in hUCB produced IL-10 and inhibited T-cell IFN-γ [1.63 vs. 0.95 stimulation ratio (SR); *p* = 0.004] and IL-4 (1.63 vs. 1.44 SR; *p* = 0.39) production. Phenotypically, hUCB Breg cells presented IgM^hi^IgD^hi^CD5^+^CD10^+^CD27^−^ markers, similar to those described in hAPB Breg cells, but they showed increased IgM concentration and decreased expression of CD22 and CD73 markers. Our work characterized the frequency, phenotype, and function of Breg in hUCB, which may contribute to understanding of immune tolerance during pregnancy, paving the way to a new approach to immune-related diseases in the fetus and the newborn.

## Introduction

Avoidance of unwanted immune responses through peripheral tolerance involves several regulatory/suppressor molecules and cell types. Regulatory B (Breg)-cells are a rare B-cell subpopulation with this regulatory/suppressor function. Several markers have been described for the detection and sorting of these cells. This is nicely reviewed by Rosser and Mauri (1). Breg cells are known to perform their suppressive action by IL-10/TGF-β production, cell-to-cell contact by CD80/86 interaction with T-cells, and CD73-dependent adenosine production (1–6). CD24^hi^CD38^hi^ and CD24^hi^CD27^+^ have been used recently for the study of Bregs in pregnant women (7).

The most studied subset of Breg cells is defined by CD24^hi^ and CD38^hi^ expression in B-cells (2, 6, 8). Phenotypically, these cells also express IgM, IgD, CD5, CD10, and CD1d (2), resembling transitional B-cells (9). Breg cells are mainly defined by their regulatory function: Mauri et al. demonstrated that the CD19^+^CD24^hi^CD38^hi^ subset is enriched in IL-10 production and can inhibit IFN-γ production (2, 10) and block T helper-cell (Th)1 and Th17 differentiation while maintaining T regulatory (Treg) cell population (6). Their implication in human immune-related diseases has been studied mostly in autoimmune (11) and allergic diseases (12, 13), persistent infections such as HIV (14), HBV (15), and *Mycobacterium tuberculosis* (16), cancer (17), transplantation and, as demonstrated recently, pregnancy (7, 12, 18–20).

Regulatory B-cells are an important player in the achievement of the tolerogenic immune state of the mother to its haploidentical fetus during pregnancy. Maternal–fetal tolerance is achieved through different mechanisms such as an increase of Treg cells, expression of CD274 (PD-L1) in the trophoblastic tissue, and an increase of Breg cells (21, 22). Early pregnancy factor enhances Treg-cell production and IL-10 and TGF-β expression in splenocytes from female mice (23). In pregnant mice, the increase in Breg is necessary to avoid immunological abortion. In fact, the transfer of Breg cells to abortion-prone mice leads to a Treg-cell increase and maintains dendritic cells in an immature state, promoting fetal–maternal tolerance (19). In humans, B-cells increase IL-10 production in response to human gonadotropic hormone from pregnant woman serum (18). Also, there is an increase of Breg during the first trimester of pregnancy that does not occur in women with spontaneous abortion (18). Moreover, women treated with rituximab, a B-cell-depleting antibody, during pregnancy presented a higher rate of first-trimester pregnancy loss (24). The role of B cells during pregnancy changes in its various stages. A decrease in CD24^hi^CD38^hi^ B cells in the third trimester of pregnancy has been described recently (7), as lower levels of IL-10 in pregnant women (25). Furthermore, there are lower BAFF levels in pregnant women suffering from preeclampsia in comparison with healthy ones; BAFF levels are higher in healthy umbilical cord blood (hUCB) than in the pregnant mother at the time of delivery (26). These data highlight the importance of B-cells, specifically Breg, in the mother’s achievement of immune tolerance during the first stages of pregnancy.

B-cell development and maturation is a complex and regulated process. In peripheral blood, we can encounter different B cell subsets that include naïve, transitional, marginal zone-like B-cells [expressing IgM, IgD, and CD27 in their membrane (27, 28)], mature B-cells, and plasmablasts (27, 29).

B-cells have been thought to be mere antibody factories for years, but it is now known that they have different functions that include cytokine production and regulation of T-cell responses. Activation status of B-cells has been studied. CD25 expression in B-cells is related with better antigen presentation, more proliferation, and an increased response to IL-2 (30). Another B-cell activation marker is CD71, the transferrin receptor. CD71 regulates the iron uptake of activated B-cells (31). Activation of B-cells is tightly modulated. CD22 is a B-cell-restricted molecule that downregulates the signal between CD19 and the BCR (32–34). The lack of this regulatory molecule provokes an increase in B10 cells in mice (35).

Along with an important anti-infection role, the immune system of the fetus must also tolerate its haploidentical mother as well as harmless antigens after delivery. To reduce the risk of alloimmune reactions between mother and fetus, APCs from the newborn selectively impair production of Th1-related cytokines (36). Although vaginal or cesarean delivery can affect leukocyte populations and plasma concentration of some cytokines (37), hUCB T-cells presented lower IFN-γ production after mitogen stimulation independently of the way of delivery (38). This regulation is partially explained by impaired IL-12 production caused by a defect in nucleosome remodeling and the repression of IL-12p35 at the chromatin level. Also, murine CD5^+^ B-cells in neonates have been described as contributing to the reduced production of IL-12 by APCs through IL-10 production in response to TLR9 stimulation (39). Recently, it was described how asthmatic mothers of infants with early allergy had an increase in transitional B-cells in the late-pregnancy period, suggesting that these cells may play a role in the Th1/Th2 bias observed in neonates (20). Furthermore, it is known that infusion of stem cells from hUCB rather than adult bone marrow enables transplantation in patients with increased donor–recipient HLA mismatch (40). Recently, it was shown that B-cell-mediated regulation was one of the possible mechanisms explaining this augmented allogenic tolerance (41).

Human Breg cells in umbilical cord blood and their implications in the achievement of tolerance are not fully characterized. This work is an attempt to further characterize the Breg cell population in cord blood of healthy neonates. We observed an increased number of CD19^+^CD24^hi^CD38^hi^ B-cells, especially among marginal zone-like B-cells, phenotypically comparable to adult Breg but with particular differences and functionally inhibiting IFN-γ and IL-4 production.

## Materials and Methods

### Blood Extraction

Eight healthy pregnant women with uneventful pregnancies and eight anonymous adult healthy controls [three males and five non-pregnant females; median age, 25.5 years (range, 21–50 years)] were enrolled in this study over a period of 2 months. hUCB from the enrolled pregnant women was drawn at the time of delivery. A total volume of 20 ml was drawn from each subject or from the hUCB into lithium-heparinized and EDTA-treated tubes.

This study was carried out in accordance with the recommendations of *Ley General de Sanidad (25/4/1986) Art. 10* with written informed consent from all subjects. All subjects gave written informed consent in accordance with the Declaration of Helsinki. The protocol was approved by the ethics committee of the Hospital Sant Joan de Déu (number of the *Comité Ético de Investigaciones* Clínicas: PIC-50-12).

### Peripheral Blood Mononuclear Cells (PBMCs) Isolation, Sorting, and Culture

Peripheral blood mononuclear cells from hUCB and healthy controls were isolated with Ficoll-Hipaque (Sigma-Aldrich, St. Louis, MO, USA) density gradient centrifugation of heparinized blood. Cells were subsequently washed three times with PBS 1× (Roche Diagnostics, Barcelona, Spain) and cultured with complete medium [RPMI (Gibco, Grand Island, NY, USA) supplemented with 10% heat-inactivated fetal calf serum (FCS; Sigma-Aldrich, St. Louis, MO, USA), 1 μg/ml penicillin, and 1 μg/ml streptomycin (Invitrogen, Grand Island, NY, USA)]. Viable cells were counted using a hemocytometer in an inverted microscope.

We explored the regulatory function of hUCB B-cells by studying CD3^−^CD19^+^CD24^hi^CD38^hi^ cell subset because although it is a heterogeneous population, most Breg cells lie in this compartment. For Th1/Th2/Th17 evaluation, coculture assays were performed in sorted PBMCs isolated from hUCB: hUCB PBMCs were sorted using the FACS-ARIA II sorter (BD Bioscience, San Jose, CA, USA). Cell populations were defined as Breg: CD3^–^CD19^+^CD24^hi^CD38^hi^, NoBreg: CD3^–^CD19^+^CD24^int^CD38^int^ (2), and T-cells: CD3^+^CD19^–^CD25^−^. These hUCB sorted cells were cocultured in a CD3 pretreated plate (24 h, 4°C, 0.5 μg/ml, clone 33-2A3 developed in HCB by Dr. R. Vilella) in three different conditions: T-cells only, T-cells and Breg at 1:1 ratio, and T-cells and NoBreg cells at 1:1 ratio. After 72 h of coculture, cells were stimulated for 5 h with PMA–ionomycin–monensin [PIM; PMA 50 ng/ml (Sigma-Aldrich, St. Louis, MO, USA) + ionomycin 1 μl/ml (Sigma-Aldrich, St. Louis, MO, USA) in the presence of monensin (GolgiStop from Th1/Th2/Th17 phenotyping kit, 4 μl for 6 ml of culture)] or left with monensin alone to stop cytokine secretion in the Golgi compartment. Cells were stained for flow cytometry.

For IL-10 detection in the supernatant of B-cell cultures, we performed positive magnetic separation of CD19^+^ cells (>98% purity). After separation, 400,000 B-cells were cultured in a 96-well plate with or without CD40L (1 μg/ml) and CpG-B (1 μg/ml). After 48 h, 100 μl of supernatant were removed and analyzed by Luminex following manufacturer’s instructions. Cells were cultured for 5 more hours in the presence of brefeldin A (BFA) alone or LPS/PMA/ionomycin and BFA. Intracellular cytokine staining (ICS) for IL-10 detection was then performed.

### Flow Cytometry

For the immunophenotype, cell staining was performed in 50 μl EDTA whole blood. For surface staining, whole blood was incubated for 15 min at room temperature (RT) with the surface antigen antibodies at proper concentration. To lyse and fix cells, cells were incubated with 2 ml of BD FACS lysing solution 1× (BD bioscience, San Jose, CA, USA) for 15 min at RT. Cells were then washed two times with FACS buffer (phosphate-buffered saline with 5% FCS, 0.5% BSA, and 0.07% NaN_3_) and acquired using a FACSCanto-II (BD Bioscience) cytometer.

T regulatory intracellular staining was performed with Treg Detection Kit (CD4/CD25/FoxP3) (Milteny Biotec, Germany) following the manufacturer’s instructions. Briefly, surface staining was performed as described above, and then cells were fixed with 500 μl of fixation buffer for 30 min at 4°C. Cells were washed two times with FACS buffer and a third time with Perm Buffer. To perform the blocking, cells were incubated with 20 μl of Perm Buffer and 5 μl of FcR Blocking Reagent for 5 min at RT. Cells were then stained with FoxP3-APC antibody for 30 min at 4°C. Finally, cells were washed with FACS buffer and acquired with the cytometer.

For detection of IL-10 production, as previously reported (21), cells were matured 48 h with CD40L (1 μg/ml; Insight genomics, Falls Church, VA, USA) and ODN-2006 Cp (USA), PMA (50 ng/mL), and ionomycin (1 μl/ml) in the presence of BFA (10 μl/ml, Sigma-Aldrich, St. Louis, MO, USA). For detection of spontaneous IL-10 production, cells were incubated for 5 h in the presence of BFA to stop cytokine secretion *via* Golgi transport and then washed with FACS Buffer and incubated with mAb anti-human surface molecules for 15 min at RT; cells were then permeabilized following “Th1, Th2, Th17 phenotyping kit^®^” (BD Bioscience, San Jose, CA, USA) protocol described below and incubated for 30 min at RT, dark with anti-IL-10 Ab. Cells were then washed with FACS buffer and acquired with the cytometer.

Intracellular Cytokine Staining for IFN-γ, IL-4, and IL-17 was performed following the “Th1, Th2, Th17 phenotyping kit^®^” (BD Bioscience, San Jose, CA, USA) protocol. Briefly, cells were first washed with FACS buffer and then fixed incubating with 600 μl of Cytofix buffer for 10 min at RT and washed twice with FACS buffer. To permeabilize them, the membrane cells were incubated with 600 μl of Perm/Wash for 15 min at RT and then incubated for 45 min with the corresponding surface and intracellular antibodies. Cells were then washed with FACS buffer and acquired with the cytometer.

Panels of direct fluorochrome-conjugated anti-human antibodies were used with the following antibodies: CD3-APCCy7, CD3-AlexaFluor750, CD4-FITC, CD4-PerCPCy5.5, CD5-APC, CD8-PerCPCy5.5, CD10-APCH7, CD16-PE, CD19-PECy7, CD19-APCCy7, CD19-BV510, CD21-FITC, CD21-PECy5.5, CD22 BV421, CD24-PerCPCy5.5, CD25-PE, CD25-APCH7, CD27-PECy7, CD38-APC, CD38-PECy7, CD45-APC, CD45-PECy7, CD56-PE, CD71-APC, CD73-BV421, CD86-FITC, CD127-PECy7, IFN-γ-FITC, IgD-PE, IgD-FITC, IgM-FITC, IgM-BV421, IL-4-APC, IL-10-PE, IL-17-PE, PD-L1-APC, TCRab-FITC, and TCRgd-PE. See Table 1 for panel and antibodies details.

**Table 1:** **Antibody panels used for umbilical cord and adult peripheral blood phenotyping**.

Panel	Antibody specificity–fluorochrome-Clone					
T–B–NK phenotype	CD3^a^	CD4^b^	CD8^a^	CD16/CD56	CD19^a^	CD45^a^
	APCCy7	FITC	PerCPCy5.5	PE	PECy7	APC
	SK7	VIT4	RPA-T8	B73/MY31	HIB19	2D1

T-subphenotype	CD3^a^	CD8^a^	CD45RA^a^	CD45RO^a^	TCRab^a^	TCRgd^a^
	APCCy7	PerCPCy5.5	PECy7	APC	FITC	PE
	SK7	RPA-T8	L48	UCHL1	WT31	11F2

B-subphenotype-1	CD19^a^	CD21^a^	CD38^a^	IgD^a^	IgM^c^	
	PECy7	PECy5	APC	PE	FITC	
	SJ25C1	B-ly4	HIT2	IA6-2	SA-DA4	

B-subphenotype-2	CD19^a^	CD27^c^	CD38^a^	IgD^a^	IgM^c^	
	PECy7	PECy7	APC	PE	FITC	
	SJ25C1	1A4CD27	HIT2	IA6-2	SA-DA4	

Regulatory T-cells	CD3^c^	CD4^b^	CD25^b^	CD127^a^		
	AlexaFluor750	FITC	PE	PECy7		
	UCHT1	VIT4	4E3	HIL-7R-M221		

Regulatory B-cells	CD19^a^	CD24^a^	CD38^a^			
	PECy7	PerCPCy5.5	APC			
	SJ25C1	ML5	HIT2			

Breg sorting	CD3^c^	CD19^a^	CD25^b^	CD24^a^	CD38^a^	
	FITC	PECy7	PE	PerCPCy5.5	APC	
	UCHT1	HIB19	4E3	ML5	HIT2	

IL-10 intracellular cytokine staining	CD19^a^	IL-10^a^				
	PECy7	PE				
	HIB19	JES3-9D7				

Th1/tTh2/Th17 phenotyping kit	CD3^a^	CD4^a^	IFNg^a^	IL4^a^	IL17^a^	
	APCCy7	PerCPCy5.5	FITC	APC	PE	
	SK7	SK3	B27	MP4-25D2	N49-653	

Breg panel 1	CD19^a^	CD24^a^	CD38^a^	CD27^c^	IgM^a^	IgD^c^
	APCCy7	PerCPCy5.5	APC	PECy7	BV421	FITC
	SJ25C1	ML5	HIT2	1A4CD27	G20-127	IADB6

Breg panel 2	CD19^a^	CD24^a^	CD38^a^	CD25^a^	CD73^a^	CD71^d^
	BV510	PerCPCy5.5	PECy7	APCH7	BV421	APC
	SJ25C1	ML5	HB7	M-A251	AD2	HI166

Breg panel 3	CD19^a^	CD24^a^	CD38^a^	CD21^a^	CD5^c^	CD10^e^
	BV510	PerCPCy5.5	PECy7	FITC	APC	APCH7
	SJ25C1	ML5	HB7	B-Ly4	BL1a	HI10a

Breg panel 4	CD19^a^	CD24^a^	CD38^a^	PD-L1^e^	CD22^e^	CD86^d^
	APCCy7	PerCPCy5.5	PECy7	APC	BV421	FITC
	SJ25C1	ML5	HB7	29E-2A3	S-HCL-1	BU63

*^a^BD Biosciences*.

*^b^Milteny Biotech*.

*^c^Beckman Coulter (Brea, CA, USA)*.

*^d^Immunotools (Friesoythe, Germany)*.

*^e^Biolegend (San Diego, CA, USA)*.

Cells were acquired on a FACS CANTO II flow cytometer (BD Bioscience, San Jose, CA, USA) within the next 2 h after cell staining. Data analysis was performed using FlowJo 7.3 software (TreeStar, Inc., Ashland, OR, USA). Absolute number of lymphocytes was determined with a hematological cell counter.

### Statistical Analysis

Statistical analysis was performed using Prism 6 software (GraphPad, La Jolla, CA, USA) and Microsoft Excel (2010). As the cohort did not show normal distribution, we performed Mann–Whitney test for all conditions. Differences between values were considered statistically significant when *p* < 0.05.

## Results

### Description of Reference Values of Leukocyte and Lymphocyte Subsets in hUCB

We first analyzed monocyte, neutrophil, and lymphocyte subset frequency. For lymphocyte subset study, we based our panels on and used the reference values of clinical guide references (27, 42–44). Values observed were within-reported reference values (27, 42, 43, 45, 46). We encountered, as expected, significant differences between healthy adult peripheral blood [healthy peripheral adult blood (hAPB), *n* = 5] and hUCB (*n* = 6). Specifically, we observed more naïve T-cell compartment (CD45RA^+^CD45RO^−^, 46.9 vs. 24.18% of T-cells, *p* = 0.004). B-cells were also enriched in the more immature subsets. There was an increase in IgD/IgM double-positive B-cells (92 vs. 62.28% of B-cells, *p* = 0.002), marginal zone-like B-cells (CD19^+^IgM^+^IgD^+^CD27^+^; 40.74 vs. 17.29% of B-cells, *p* = 0.002), naïve B-cells (CD19^+^IgD^+^CD27^−^; 47.7 vs. 17.4% of B-cells; *p* = 0.0002), and transitional B-cells (CD19^+^CD38^+^IgM^hi^; 54 vs. 18.78% of B-cells, *p* = 0.006) (Table 1; Table 2; Figure S1 in Supplementary Material).

**Table 2:** **Evaluation of different leukocyte and lymphocyte populations and subpopulations in hUCB vs hAPB**.

Population	hUCB		hAPB		*p*
	Mean	SEM	Mean	SEM	
**% of leukocytes**					

Monocytes	7.54	1.39	5.77	1.25	0.02
Lymphocytes	29.63	7.19	22.42	4.69	0.11
Neutrophils	41.31	9.47	52.28	8.19	0.04

**% of lymphocytes**					

T lymphs	65.96	4.33	74.88	1.79	0.22
B lymphs	17.81	2.03	13.76	1.36	0.23
NK cells	13.80	3.20	9.47	2.19	0.41
NKT-cells	1.62	0.23	6.16	1.15	0.002
CD3^+^CD4^+^	46.70	3.47	45.54	2.50	0.62
CD3^+^CD8^+^	17.21	1.44	23.56	1.79	0.03
CD3^+^CD4/8^−^	1.30	0.15	5.11	1.13	0.01

**% of T-cells**					

TCR_αβ_	93.85	0.4822	89.3	1.202	0.009
TCR_γd_	1.90	0.3044	7.32	1.976	0.03
CD45RA^+^CD45RO^−^	46.9	2.321	24.18	5.006	0.004
CD8^+^CD45RA^+^CD45RO^−^	16.2	1.236	10.71	2.866	0.25
CD8^−^CD45RA^+^CD45RO^−^	30.73	2.333	13.45	2.866	0.009
CD45RO^+^	52.65	2.301	75.72	5.035	0.004
CD8^+^CD45RO^+^	9.765	1.174	19.14	2.185	0.004
CD8^−^CD45RO^+^	42.9	1.784	56.51	4.081	0.004
CD45RA^+^CD45RO^+^	44.87	2.091	37.94	5.966	0.66
CD8^+^CD45RA^+^CD45RO^+^	10	1.147	17.79	1.95	0.004
CD8^−^CD45RA^+^CD45RO^+^	35.18	1.895	20.1	4.82	0.02
CD45RA^−^CD45RO^−^	8.048	0.7985	37.78	2.205	0.004
CD8^+^CD45RA^−^CD45RO^−^	0.1217	0.03497	1.35	0.3404	0.004
CD8^−^CD45RA^−^CD45RO^−^	7.923	0.7755	36.41	2.145	0.004
Treg cells	0.52	0.13	0.23	0.08	0.13

**% of B-cells**					

IgD^+^IgM^+^	92	1.304	62.28	4.617	**0.002**
Marginal zone-like B-cells	40.74	2.464	17.29	3.669	**0.002**
Naïve B-cells	47.7	3.487	4.046	0.7584	**0.002**
Transitional B-cells	54	4.88	18.78	2.761	**0.002**
IgD^+^IgM^−^	3.638	1.649	14	2.875	**0.006**
IgD^−^IgM^−^	3.903	0.5775	1.639	0.3791	**0.003**
Switched memory B-cells	2.574	0.5774	63.8	5.043	**0.002**
IgD^−^IgM^+^	0.4338	0.08744	3.603	0.7951	**0.0003**
CD38^low^CD21^low^ B-cells	0.0475	0.02541	2.377	0.5152	**0.0003**
Plasmablasts	1.391	0.2774	3.92	0.8957	**0.02**

*hUCB, human umbilical cord blood; hAPB, human adult peripheral blood; p, significance value; marginal zone-like B-cells, CD19^+^IgM^+^IgD^+^CD27^+^; switched memory B-cells, CD19^+^IgM^−^IgD^−^CD27^+^; naïve B-cells, CD19^+^IgD^+^CD27^−^; transitional B-cells, CD19^+^IgM^hi^CD38^hi^; plasmablasts, CD19^+^IgM^+^CD38^low^*.

### Breg Cells Are Present at Higher Frequency in hUCB than in hAPB

**Figure 1:**
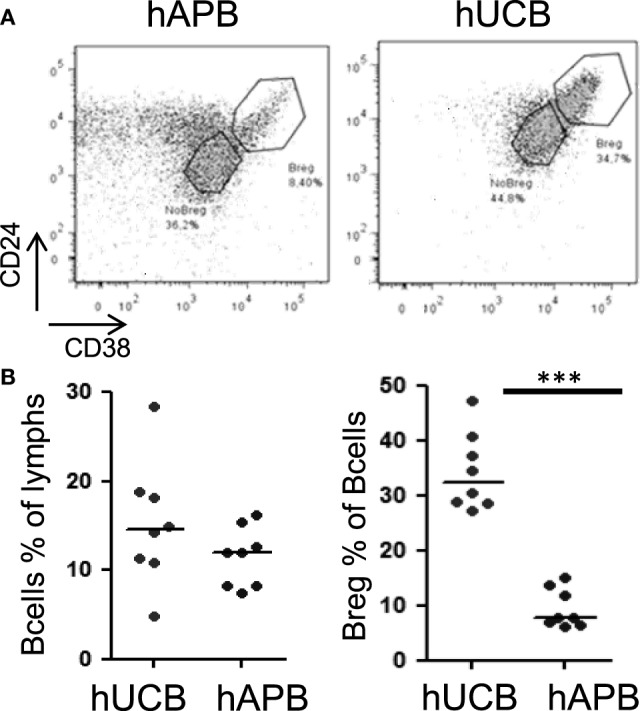
**hUCB is enriched in Breg population when compared with hAPB**. **(A)** Breg gating strategy and representative example of hAPB/hUCB Breg and NoBreg cells. **(B)** Frequency of B-cells and Breg cells among B-cells in hAPB (*n* = 8) and hUCB (*n* = 8). hUCB, human umbilical cord blood; hAPB, human adult peripheral blood; Breg, B regulatory cell; NoBreg, non-regulatory B-cell. ***p < 0.001.

**Figure 2:**
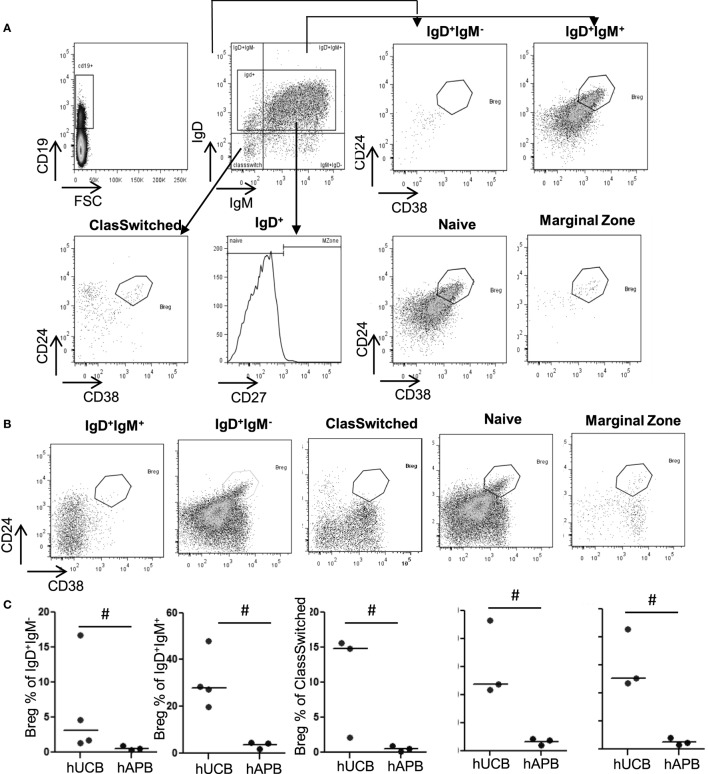
**Breg frequency is significantly higher in hUCB than in hAPB among marginal zone-like, naïve, and IgD^+^IgM^+^ cells**. **(A)** Gating strategy and representative hUCB example. **(B)** Representative hAPB example. **(C)** Breg percentage in different B-cell populations in hUCB (*n* = 3) and hAPB (*n* = 4). hUCB, human umbilical cord blood; hAPB, human adult peripheral blood; Breg, regulatory B-cell; NoBreg, non-regulatory B-cell; MZ, marginal zone-like. *p < 0.05.

There was an increased frequency of Breg cells among total CD19^+^CD24^hi^CD38^hi^ B-cells in hUCB (34.39% ± 2.49 of B-cells in hUCB, 9.49 ± 1.27% of B-cells in hAPB; mean ± SD; *p* = 0.0002) (Figure 1). After evaluating the presence of Breg cells among total B-cells, we studied Breg cells in different B-cell subsets (Figures 2A,B) in both hUCB and hAPB. Studied B-cell subsets were defined as (i) CD19^+^IgM^+^IgD^+^, (ii) CD19^+^IgM^−^IgD^+^, (iii) naïve cells (CD19^+^IgD^+^CD27^−^), (iv) marginal zone-like cells (CD19^+^IgD^+^CD27^+^), and (v) class-switched cells (CD19^+^IgM^−^IgD^−^). In both hUCB and hAPB, CD19^+^CD24^hi^CD38^hi^ Breg cells were present in all the above-described B-cell subsets. Nevertheless, the marginal zone-like B-cell subset was the most enriched in Breg and showed the most marked difference between hUCB and hAPB (60.8 vs. 4.94%; *p* = 0.1), compared to the other subsets: naïve subset (30.58 vs. 3.26%; *p* = 0.1), IgD^+^IgM^+^ B-cells (30.76 vs. 3.39%; *p* = 0.057), IgD^+^IgM^−^ B-cells (6.02 vs. 0.51% of B-cells; *p* = 0.057), and class-switched B-cells (10.8 vs. 0.49% of B-cells; *p* = 0.1) (Figure 2C).

### IL-10 Production at Baseline Is Increased in hUCB Compared with hAPB, in Both Breg and Non-Breg Compartments

**Figure 3:**
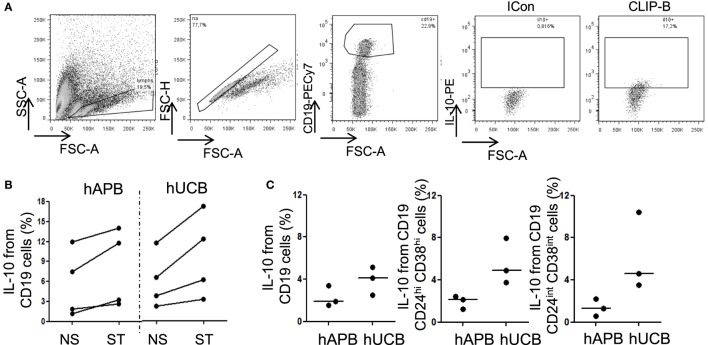
**Breg cells from hUCB can produce IL-10 after stimulation and spontaneously**. **(A)** Intracellular IL-10 staining in one representative hUCB example in CD40L + CPG maturation + CLIP-B stimulation condition. **(B)** IL-10 intracellular staining at 48 h of maturation with CD40L plus GPG followed by non-stimulation or stimulation with CLIP-B in hUCB (*n* = 4) and healthy peripheral adult blood (hAPB) (*n* = 4). **(C)** B, Breg, and NoBreg cells spontaneous production of IL-10 after 5 h incubation in the presence of BFA B in hUCB (*n* = 3) and hAPB (*n* = 3). CLIP-B, CpG, LPS, PMA, and ionomycin after 5 h in the presence of BFA; BFA, brefeldin A; hAPB, human adult peripheral blood; hUCB, human umbilical cord blood; Breg, regulatory B-cells; NoBreg, non-regulatory B-cell. **p < 0.01.

Human Breg cannot be defined solely based on a phenotype composed of conventional B-cell markers. Analyses of Breg functionality are necessary to confirm the regulatory function of this population. The capacity to produce IL-10 is an important functional item that defines CD19^+^CD24^hi^CD38^hi^ Breg cells (2–4), although it is not unique to this cell type (47, 48). We observed that hUCB B-cells produced spontaneously (by incubating cells for 5 h in the presence of BFA) higher levels of IL-10 than hAPB, but this difference was no longer observed after stimulation (Figure 3; Figure S3 in Supplementary Material). When we analyzed the different B-cell subsets, we observed that both Breg and NoBreg [defined as CD19^+^CD24^int^CD38^int^ (2)] from hUCB showed a tendency toward a spontaneously higher IL-10 production than hAPB (3.93 vs. 2.29%; *p* = 0.2 for B-cells; 5.54 vs. 1.92%; *p* = 0.1 for Breg and 6.15 vs. 1.35%, *p* = 0.1 for NoBreg; Figure 3; Table SII in Supplementary Material), suggesting an important role for IL-10 from B-cells in hUCB.

### hUCB Breg Inhibit IFN-γ and IL-4 Production by T-Cells When Cocultured

**Figure 4:**
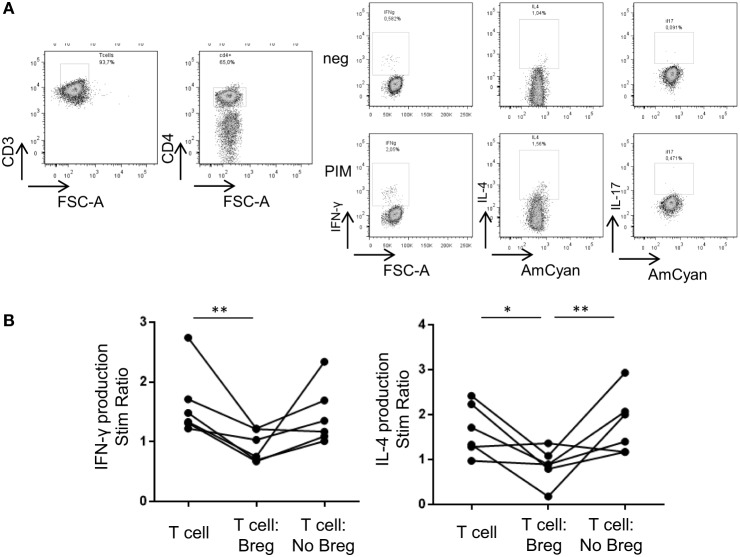
**Breg cells from hUCB inhibit IFN-γ and IL-4 production by T-cells when cocultured**. **(A)** Intracellular cytokine staining in one representative hUCB example of a T-cell-only culture with PIM stimulation. **(B)** hUCB Breg and NoBreg effect on T-cell SR (stimulated/basal) after 72 h coculture plus 5 h stimulation with PIM at 1:1 ratio (T-cells alone, T-cells:Breg, T-cells:NoBreg) in a CD3-coated plate (*n* = 6). SR, stimulation ratio; PIM, PMA and ionomycin in the presence of monensin; hAPB, human adult peripheral blood; hUCB, human umbilical cord blood; Breg, Regulatory B cells; NoBreg, non-regulatory B-cells. **p < 0.005.

To corroborate the suppressive capacity of phenotypically described hUCB Breg, we performed T:Breg and T:NoBreg coculture assays in hUCB sorted PBMCs. We analyzed IFN-γ, IL-4, and IL-17 production by T-cells after 72 h coculture in the presence of plate-bound CD3 with or without hUCB Breg (as defined by CD19^+^CD24^hi^CD38^hi^) or NoBreg cells (as defined by CD19^+^CD24^int^CD38^int^), with PMA–ionomycin in the presence of monensin during the previous 5 h. We observed a statistically significant inhibition of IFN-γ stimulation ratio (SR) by T-cells when cocultured with Breg cells [1.63 vs. 0.95 SR; *p* = 0.004]. This was not observed when cocultured with NoBreg cells (1.63 vs. 1.44 SR; *p* = 0.39). For IL-4, we observed a significant decrease in the SR in the T-cell-Breg coculture condition in comparison with T-cells alone (1.66 vs. 0.86 SR; *p* = 0.02) and T-cells:NoBreg (0.86 vs. 1.79 SR; *p* = 0.009). We cannot state the effect of Breg cells in the IL-17 production as we cannot clearly detect its production after PIM stimulation (Figure 4; Table SIII in Supplementary Material). These data demonstrated that hUCB Breg cells play a regulatory function by inhibiting IFN-γ production by T-cells when cocultured.

### hUCB Breg Show Phenotypical Differences with hAPB-Described Breg

**Figure 5:**
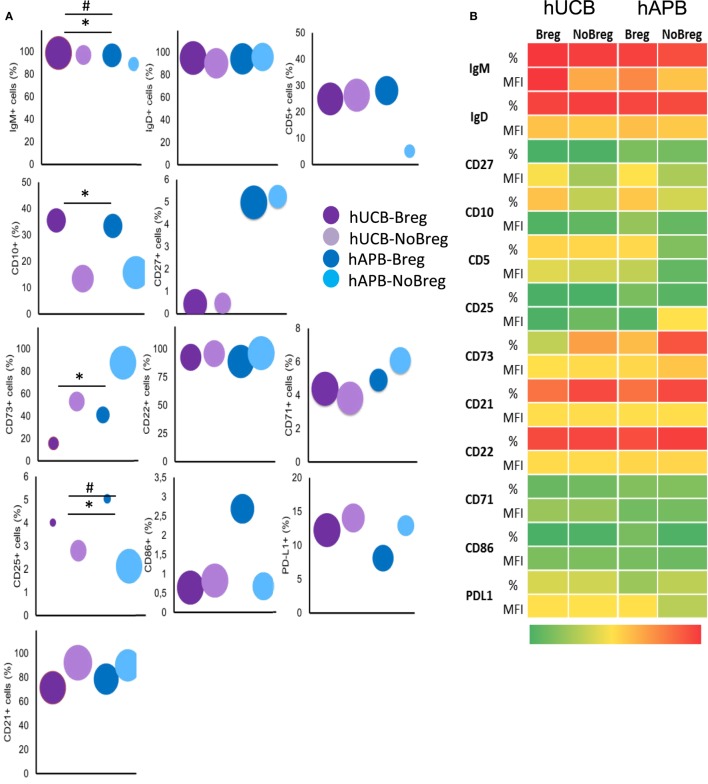
**hUCB Breg cell-defining phenotype corresponds with that previously described for Bregs**. **(A)** Frequency of known Breg cell markers and others among Breg and NoBreg cells. Y axis value corresponds to % of positive cells, and area of the circle corresponds to the MFI of the whole population. **(B)** Heatmap of % and MFI of the different T-cell markers in Bregs and NoBregs from hAPB (*n* = 3) and hUCB (*n* = 4), with green being the lowest values and red the highest. hUCB, human umbilical cord blood; hAPB, human adult peripheral blood; Breg, B regulatory cell; NoBreg, non-regulatory B-cell; MFI, mean fluorescence intensity. * and ^#^ denote MFI and frequency statically significant differences, respectively. *^/#^*p* < 0.05, **^/##^*p* < 0.01, and ***^/###^*p* < 0.001.

In concordance with the reported phenotype (2), hAPB Breg were IgM^hi^IgD^hi^CD5^+^CD10^+^CD27^low^. hUCB Breg presented a similar phenotype but with some differences: hUCB Breg showed more IgM per cell [mean fluorescence intensity (MFI); 13,380 vs. 7,716; *p* = 0.057] and a diminished frequency of CD27^+^ cells (0.65 vs. 6.11%; *p* = 0.057). hUCB Breg were then IgM^hi+^IgD^hi^CD5^+^CD10^+^CD27^−^. This difference in IgM and CD27 expression was extendable to, but less marked in, NoBreg cell compartment. Concretely, NoBreg cells in hUCB presented a IgM^+^IgD^+^CD5^−^CD10^−^CD27^low^ phenotype, which compared to the adult had higher MFI of IgM (4,953 vs. 2,706; *p* = 0.057), an increased CD5 frequency (26.01 vs. 6.59%; *p* = 0.057) and MFI (202.5 vs. 39.88; *p* = 0.057), and a diminished frequency of CD27^+^ cells (0.92 vs. 5.34%; *p* = 0.1) (Figure 5; Table SIV and Figure S2 in Supplementary Material).

### hUCB Breg Present Lower Membrane Expression of CD22 than hAPB Breg

In addition to the previously described markers, we studied others related to the activation and maturation state of B-cells. For each marker, we compared hUCB Breg cells with NoBreg cells and with their adult equivalents. For the activation status of Breg cells, we studied CD22, CD25, CD71, CD86, and PD-L1 markers. In hUCB, we observed an increase in frequency and a decrease in MFI of CD25 in Breg cells when compared with NoBreg cells (4.37 vs. 2.78%; *p* = 0.03 and MFI: 7.46 vs. 54.33; *p* = 0.03). We observed no differences in CD71, CD22, CD86, and PD-L1 expression between hUCB Breg and NoBreg cells. Interestingly, there were differences between Breg from hUCB and hAPB: there was a decreased CD22 expression in hUCB (MFI: 800.9 vs. 1,293; *p* = 0.057) and a lower CD86 frequency (0.58 vs. 5.8%; *p*: 0.057). These differences did not reach statistical significance possibly due to the high variability observed in adults (Figure 5; Table SIV in Supplementary Material; Figure S2 in Supplementary Material). This variability may account for the different antigenic stimulations occurring during the lifespan of a person and the divergence of this experience increasing over the years.

### CD73 Ectonucleotidase Seems Not to Be a Mechanism of Regulation in hUCB

CD73 is an ectonucleotidase that produces adenosine-inhibiting T-cell responses in an IL-10-independent way that has been related with regulatory functions of B-cells (5). To explore the presence of an IL-10-independent suppression mechanism in hUCB, we studied CD73 expression in the membrane of Breg and NoBreg cells in both hUCB and hAPB. There was a lower CD73 expression frequency in Breg cells compared with NoBregs, in both hUCB (13.45 vs. 50.83%; *p* = 0.03) and hAPB (37.98 vs. 85.7%; *p* = 0.1). Of note, the frequency observed in hUCB Breg was lower than hAPB (*p* = 0.057). This fact suggests that the IL-10-independent suppression by CD19^+^CD24^hi^CD38^hi^ Breg cells is not related to CD73 expression (Figure 5; Table SIV in Supplementary Material; Figure S2 in Supplementary Material).

## Discussion

This study presents the functional and phenotypical characterization of the increased Breg cell subset in hUCB defined as CD19^+^CD24^hi^CD38^hi^ cells. Bregs were present with greater frequency in hUCB and were most markedly observed in marginal zone-like B-cells. At a functional level, hUCB B-cells (including both Breg and NoBreg) produced higher levels of IL-10 spontaneously. hUCB Bregs demonstrated their regulatory capacity by differentially inhibiting IFN-γ and IL-4 production by T-cells when cocultured. Phenotypically, hUCB Bregs presented a similar phenotype to that described in hAPB with an increased quantity of IgM per cell and a diminished frequency of CD27 and with less CD22 in their membrane. Finally, hUCB Bregs also presented lower expression levels of the IL-10-independent inhibitor CD73. To our knowledge, this is the second attempt to characterize the population of CD19^+^CD24^hi^CD38^hi^ cells in human cord blood. The first attempt was recently reported (41), but the phenotype of hUCB Breg was not fully characterized.

Healthy umbilical cord blood marginal zone-like B-cells presented the greatest difference in proportion of Breg cells when compared with adults. Circulating marginal zone B-cells are representative of splenic marginal zone B-cells, having a prediversified immunoglobulin repertoire and initiating T-cell-independent responses through TLRs as activation signals. Their responses are mainly directed against encapsulated bacteria, including commensal microbiota (49–51). Marginal zone B-cells also play a role in normal pregnancy development (52), and it is known that antiencapsulated bacteria responses are lower in infants and young children (36). As TLRs activate Bregs (2, 3), the greater proportion of marginal zone-like B-cells observed might indicate increased cooperation in regulatory function after encapsulated bacterial stimulation. Also, we observed lower levels of CD22 in the membrane of hUCB Bregs, suggesting a lower activation threshold (32), which could be of importance in the rapid abrogation of unwanted responses to commensal bacteria. During the first contacts with the extrauterine environment and the adoption of microbiota, we might hypothesize the increased proportion of Breg cells among marginal zone B-cells to be one of the mechanisms by which the neonatal immune system protects itself from an exacerbated response to the new range of antigens it is exposed to.

We observed that all B-cells, Breg, and NoBreg, from hUCB spontaneously produced more IL-10 than their adult counterparts, but there were no significant differences between Breg and NoBreg hUCB cells. It has previously been observed that CD19^+^CD24^hi^CD38^hi^ and CD19^+^CD24^int^CD38^in^ T-cells from hUCB are capable of producing similar amounts of IL-10 after stimulation (41). Unlike other recently published studies (41), we observed a significant inhibition of IFN-γ production only when T-cells and Bregs were cocultured. We speculate that this discrepancy could be due to differences in the experimental design. We performed a coculture with either Breg or NoBreg with CD3^+^CD25^−^ T-cells for 72 h in the presence of plate-bound CD3 and, following that, a restimulation for ICS. Sarvaria et al. cocultured CD4^+^ cells with either Breg or NoBreg cells, stimulated them with anti-CD3/CD28 for 96 h, and then analyzed the concentration of inflammatory cytokines in the supernatant (41).

The mechanism by which hUCB Breg cells perform their regulatory function is unclear. We observed a differential regulatory activity of Breg and NoBreg cells spontaneously producing similar amounts of IL-10. It is known that Breg cells can carry out part of their functions *via* IL-10-independent mechanisms, such as IL-35 and TGF-β secretion and cell-to cell-contact (1, 6). Immune regulation by hUCB B-cells seems to be partially mediated by (i) IL-10 production, as seen in IL-10-blockade experiments and (ii) cell-to-cell direct contact (mediated by CD80/CD86), but independently of TGF-β (41). The mechanisms by which hAPB Bregs perform their suppressive activity have been further studied (5–7, 53), but more investigation is needed to understand whether there are other mechanisms in hUCB Breg cell regulation of T-cells.

We observed an increased quantity of IgM per cell in hUCB Breg cells compared to NoBreg cells and hAPB Breg cells, which could have implications in the mechanism of action of these Breg cells. Naturally occurring IgM antileukocyte autoantibodies (IgM-ALA) are antibodies with suppressor capacity that can inhibit T-cell activation and chemotaxis (54, 55). IgM-ALA antibodies are present at birth (55), and they inhibit pro-inflammatory cells from producing IFN-γ and IL-17 in response to alloantigens in mice (54). Altogether, we speculate that this could be a new Breg IL-10-independent mechanism for their regulatory function that could be of special importance during pregnancy and the postnatal period. More research should be done to evaluate whether these are true regulatory mechanisms of hUCB Breg function.

Healthy umbilical cord blood Breg cells presented diminished CD22 expression, suggesting a lower activation threshold. CD22 modulates the BCR signal and prevents the hyperactivation of B-cells upon stimulation (32–34). CD22 knock-out mice presented increased circulating IgM and with more IL-10-competent T-cells (35) and Breg cells CD1d^hi^CD5^+^ (56) in the spleen. These B-cells have the capacity to inhibit the IgG response to non-self and self-antigens (35). Also, *in vivo* treatment of mice with anti-CD22 mAb depletes spleen B10 and marginal zone cells in mice (57). We hypothesize that the lower level of CD22 in hUCB Breg is one of the explanations for the increased frequency of Breg among marginal zone-like B-cells. Although there is a Breg cell subset characterized by high expression of CD25 in the membrane of B-cells (53), we did observe a decrease in the presence of this receptor. CD25^hi^ Bregs described by Kessel et al. also presented high levels of CD86 and CD27 (53), suggesting that these two Breg cell groups have a convergent function but are not the same subsets.

Regulatory B cell population can be of importance in the Th1/Th2 bias observed in neonates (39). The neonate immune system has the enormous challenge of modulating the migration from the sterile uterus to the real world (36). During pregnancy, the fetus tolerates the mother’s allogenicity, dealing after birth with a huge range of antigens and microorganisms, pathogenic or not (36, 39). We hypothesized that, as CD5^+^ murine B-cells contribute to the reduced production of IL-12 by APCs (58), increased hUCB Breg contributes to the limited Th1 response observed in neonates inhibiting IFN-γ production. It has been observed that B-cells recovered from patients after stem cell transplantation without GvHD had increased levels of IL-10-producing B-cells, suggesting the inhibitory role of these cells (41). Also, CD24^hi^CD38^hi^ B-cells have been studied during pregnancy, and it has been shown that they correlate with Treg cell numbers during pregnancy and that they increase postpartum (7, 12). There was an increase in transitional B-cells (IgM^hi^CD38^hi^) in asthmatic pregnant women whose progeny developed allergy signs before 6 months of age (20). It would be of interest to confirm whether these cells express high levels of CD24, thus producing one more clue to the role of Breg cells in the Th1/Th2 bias observed in neonates.

Availability of hUCB samples is the major limitation of our study. However, it should be mentioned that in spite of the small number of samples, the same findings were consistently found in each of the samples analyzed, which strengthens the results.

Finally, we show that Breg cells were not only present in hUCB but also present at an increased frequency compared with hAPB, especially among marginal zone-like B-cells, and that they have the capacity to produce more IL-10 already at baseline while developing their regulatory function by inhibiting T-cell IFN-γ and IL-4 production, among others, thereby likely contributing to the limited Th1 response observed in neonates at delivery. The phenotypical differences observed between hUCB and hAPB Bregs lead us to construct hypotheses about the mechanisms of tolerance achievement in the fetus. Understanding of how immune tolerance to the newborn’s haploidentical mother and to harmless antigens is achieved is of the utmost importance to further advance knowledge and treatment of immune-related diseases in the fetus and the newborn. However, more investigation with neonatal hUBC Breg cells is required to decipher their regulatory mechanisms and their role in special pregnancy conditions such as chronic autoimmune disease, HIV infection, and premature delivery.

## Author Contributions

AE-S performed the experiments, designed the study, and wrote the paper; IT and AD-M performed the experiments and revised the manuscript; and AP-M, JY, MJ, and LA designed the experiments and wrote and revised the manuscript.

## Conflict of Interest Statement

The authors declare that the research was conducted in the absence of any commercial or financial relationships that could be construed as potential conflicts of interest.

## Abbreviations

BFA, brefeldin A; Breg, B regulatory cell; FCS, fetal calf serum; hAPB, human adult peripheral blood; hUCB, human umbilical cord blood; ICS, intracellular cytokine staining; NoBreg, non-regulatory B-cell; PIM, PMA–ionomycin–monensin; RT, room temperature; SR, stimulation ratio; Th, T helper cell; Treg, T regulatory cell.

## References

[1] Rosser EC Mauri C Regulatory B-cells: origin, phenotype, and function. Immunity (2015) 42:607–12. 10.1016/j.immuni.2015.04.005 25902480

[2] Blair PA Noreña LY Flores-Borja F Rawlings DJ Isenberg DA Ehrenstein MR CD19(+)CD24(hi)CD38(hi) B-cells exhibit regulatory capacity in healthy individuals but are functionally impaired in systemic lupus erythematosus patients. Immunity (2010) 32:129–40. 10.1016/j.immuni.2009.11.009 20079667

[3] Iwata Y Matsushita T Horikawa M Dilillo DJ Yanaba K Venturi GM Characterization of a rare IL-10-competent B-cell subset in humans that parallels mouse regulatory B10 cells. Blood (2011) 117:530–41. 10.1182/blood-2010-07-294249 20962324 PMC3031478

[4] Goode I Xu H Ildstad ST Regulatory B-cells: the new “it” cell. Transplant Proc (2014) 46:3–8. 10.1016/j.transproceed.2013.08.075 24216174

[5] Kaku H Cheng KF Al-Abed Y Rothstein TL A novel mechanism of B-cell-mediated immune suppression through CD73 expression and adenosine production. J Immunol (2014) 193:5904–13. 10.4049/jimmunol.1400336 25392527 PMC4321875

[6] Flores-Borja F Bosma A Ng D Reddy V Ehrenstein MR Isenberg DA CD19^+^CD24^hi^CD38^hi^ B-cells maintain regulatory T-cells while limiting TH1 and TH17 differentiation. Sci Transl Med (2013) 5:173ra23 10.1126/scitranslmed.3005407 23427243

[7] Lima J Martins C Leandro MJ Nunes G Sousa M-J Branco JC Characterization of B-cells in healthy pregnant women from late pregnancy to post-partum: a prospective observational study. BMC Pregnancy Childbirth (2016) 16:139 10.1186/s12884-016-0927-7 27267973 PMC4895979

[8] Mauri C Ehrenstein MR The “short” history of regulatory B-cells. Trends Immunol (2008) 29:34–40. 10.1016/j.it.2007.10.004 18289504

[9] Sims GP Ettinger R Shirota Y Yarboro CH Illei GG Lipsky PE Identification and characterization of circulating human transitional B-cells. Blood (2005) 105:4390–8. 10.1182/blood-2004-11-4284 15701725 PMC1895038

[10] Khoder A Sarvaria A Alsuliman A Chew C Sekine T Cooper N Regulatory B-cells are enriched within the IgM memory and transitional subsets in healthy donors but are deficient in chronic GVHD. Blood (2014) 124:2034–45. 10.1182/blood-2014-04-571125 25051962 PMC4186534

[11] Carter NA Rosser EC Mauri C Interleukin-10 produced by B-cells is crucial for the suppression of Th17/Th1 responses, induction of T regulatory type 1 cells and reduction of collagen-induced arthritis. Arthritis Res Ther (2012) 14:R32 10.1186/ar3736 22315945 PMC3392827

[12] Lima J Nunes G Lm B Martins C Regulatory T and B cells in asthmatic women: variations from pregnancy to postpartum Treg and Breg: pregnancy to postpartum. J Investig Allergol Clin Immunol (2017) 27:46–57. 10.18176/jiaci.0086 28211345

[13] Noh J Noh G Lee SJ Lee JH Kim A Kim HS Tolerogenic effects of interferon-gamma with induction of allergen-specific interleukin-10-producing regulatory B-cell (Br1) changes in non-IgE-mediated food allergy. Cell Immunol (2012) 273:140–9. 10.1016/j.cellimm.2011.12.006 22336594

[14] Siewe B Stapleton JT Martinson J Keshavarzian A Kazmi N Demarais PM Regulatory B-cell frequency correlates with markers of HIV disease progression and attenuates anti-HIV CD8+ T-cell function in vitro. J Leukoc Biol (2013) 93:811–8. 10.1189/jlb.0912436 23434518 PMC3629440

[15] Das A Ellis G Pallant C Lopes AR Khanna P Peppa D IL-10-producing regulatory B-cells in the pathogenesis of chronic hepatitis B virus infection. J Immunol (2012) 189:3925–35. 10.4049/jimmunol.1103139 22972930 PMC3480715

[16] Zhang M Zheng X Zhang J Zhu Y Zhu X Liu H CD19(+)CD1d(+)CD5(+) B-cell frequencies are increased in patients with tuberculosis and suppress Th17 responses. Cell Immunol (2012) 274:89–97. 10.1016/j.cellimm.2012.01.007 22361174

[17] Han S Feng S Ren M Ma E Wang X Xu L Glioma cell-derived placental growth factor induces regulatory B-cells. Int J Biochem Cell Biol (2014) 57:63–8. 10.1016/j.biocel.2014.10.005 25450457

[18] Rolle L Memarzadeh Tehran M Morell-García A Raeva Y Schumacher A Hartig R Cutting edge: IL-10-producing regulatory B-cells in early human pregnancy. Am J Reprod Immunol (2013) 70:448–53. 10.1111/aji.12157 24118333

[19] Jensen F Muzzio D Soldati R Fest S Zenclussen AC. Regulatory B10 cells restore pregnancy tolerance in a mouse model. Biol Reprod (2013) 89:90. 10.1095/biolreprod.113.110791 23986569

[20] Martins C Lima J Nunes G Borrego LM Pregnancy alters the circulating B-cell compartment in atopic asthmatic women, and transitional B-cells are positively associated with the development of allergy manifestations in their progeny. Am J Reprod Immunol (2016) 76:465–74. 10.1111/aji.12595 27778417

[21] Muzzio D Zygmunt M Jensen F The role of pregnancy-associated hormones in the development and function of regulatory B-cells. Front Endocrinol (2014) 5:39 10.3389/fendo.2014.00039 PMC3978254 24744750

[22] Guleria I Sayegh MH Maternal acceptance of the fetus: true human tolerance. J Immunol (2007) 178:3345–51. 10.4049/jimmunol.178.6.3345 17339426

[23] Chen Q Zhu X Chen R Liu J Liu P Hu A Early pregnancy factor enhances the generation and function of CD4CD25 regulatory T-cells. Tohoku J Exp Med (2016) 240:215–20. 10.1620/tjem.240.215 27840373

[24] Chakravarty EF Murray ER Kelman A Farmer P. Pregnancy outcomes after maternal exposure to rituximab. Blood (2011) 117:1499–506. 10.1182/blood-2010-07-295444 21098742

[25] Power LL Popplewell EJ Holloway JA Diaper ND Warner JO Jones CA. Immunoregulatory molecules during pregnancy and at birth. J Reprod Immunol (2002) 56:19–28. 10.1016/S0165-0378(01)00146-2 12106881

[26] Bienertova-Vasku J Zlamal F Tomandl J Hodicka Z Novak J Splichal Z The presence of B-cell activating factor (BAFF) in umbilical cord blood in both healthy and pre-eclamptic pregnancies and in human breast milk. J Reprod Immunol (2015) 109:89–93. 10.1016/j.jri.2014.12.003 25656062

[27] Piatosa B Wolska-Kuśnierz B Pac M Siewiera K Gałkowska E Bernatowska E B-cell subsets in healthy children: reference values for evaluation of B-cell maturation process in peripheral blood. Cytometry B Clin Cytom (2010) 78:372–81. 10.1002/cyto.b.20536 20533385

[28] Weller S Braun MC Tan BK Rosenwald A Cordier C Conley ME Human blood IgM “memory” B-cells are circulating splenic marginal zone B-cells harboring a prediversified immunoglobulin repertoire. Blood (2004) 104:3647–54. 10.1182/blood-2004-01-0346 15191950 PMC2590648

[29] Leandro MJ. B-cell subpopulations in humans and their differential susceptibility to depletion with anti-CD20 monoclonal antibodies. Arthritis Res Ther (2013) 15(Suppl 1):S3. 10.1186/ar3908 23566754 PMC3624669

[30] Amu S Strömberg K Bokarewa M Tarkowski A Brisslert M. CD25-expressing B-lymphocytes in rheumatic diseases. Scand J Immunol (2007) 65:182–91. 10.1111/j.1365-3083.2006.01889.x 17257224

[31] van de Veen W Stanic B Yaman G Wawrzyniak M Söllner S Akdis DG IgG4 production is confined to human IL-10-producing regulatory B-cells that suppress antigen-specific immune responses. J Allergy Clin Immunol (2013) 131:1204–12. 10.1016/j.jaci.2013.01.014 23453135

[32] Moyron-Quiroz JE Partida-Sánchez S Donís-Hernández R Sandoval-Montes C Santos-Argumedo L. Expression and function of CD22, a B-cell restricted molecule. Scand J Immunol (2002) 55:343–51. 10.1046/j.1365-3083.2002.01063.x 11967115

[33] Nitschke L. The role of CD22 and other inhibitory co-receptors in B-cell activation. Curr Opin Immunol (2005) 17:290–7. 10.1016/j.coi.2005.03.005 15886119

[34] Tedder TF Poe JC Haas KM. CD22: a multifunctional receptor that regulates B lymphocyte survival and signal transduction. Adv Immunol (2005) 88:1–50. 10.1016/S0065-2776(05)88001-0 16227086

[35] Poe JC Smith SH Haas KM Yanaba K Tsubata T Matsushita T Amplified B lymphocyte CD40 signaling drives regulatory B10 cell expansion in mice. PLoS One (2011) 6:e22464. 10.1371/journal.pone.0022464 21799861 PMC3143148

[36] Levy O. Innate immunity of the newborn: basic mechanisms and clinical correlates. Nat Rev Immunol (2007) 7:379–90. 10.1038/nri2075 17457344

[37] Almanzar G Schönlaub J Hammerer-Lercher A Koppelstaetter C Bernhard D Prelog M. Influence of the delivery modus on subpopulations and replication of lymphocytes in mothers and newborns. Early Hum Dev (2015) 91:663–70. 10.1016/j.earlhumdev.2015.09.010 26513626

[38] Thornton CA Capristo CC Power LL Holloway JA Popplewell EJ Diaper ND The effect of labor on neonatal T-cell phenotype and function. Pediatr Res (2003) 54:120–4. 10.1203/01.PDR.0000069704.25043.BA 12672906

[39] Holt PG Jones CA The development of the immune system during pregnancy and early life. Allergy (2000) 55:688–97. 10.1034/j.1398-9995.2000.00118.x 10955693

[40] Ruggeri A Paviglianiti A Gluckman E Rocha V. Impact of HLA in cord blood transplantation outcomes. HLA (2016) 87:413–21. 10.1111/tan.12792 27060588

[41] Sarvaria A Basar R Mehta RS Shaim H Muftuoglu M Khoder A IL-10+ regulatory B cells are enriched in cord blood and may protect against GVHD after cord blood transplantation. Blood (2016) 128:1346–1361. 10.1182/blood-2016-01-695122 27439912 PMC5016705

[42] Schatorjé EJ Gemen EF Driessen GJ Leuvenink J van Hout RW van der Burg M Age-matched reference values for B-lymphocyte subpopulations and CVID classifications in children. Scand J Immunol (2011) 74:502–10. 10.1111/j.1365-3083.2011.02609.x 21815909

[43] Schatorjé EJ Gemen EF Driessen GJ Leuvenink J van Hout RW de Vries E Paediatric reference values for the peripheral T-cell compartment. Scand J Immunol (2012) 75:436–44. 10.1111/j.1365-3083.2012.02671.x 22420532

[44] Wehr C Kivioja T Schmitt C Ferry B Witte T Eren E The EUROclass trial: defining subgroups in common variable immunodeficiency. Blood (2008) 111:77–85. 10.1182/blood-2007-06-091744 17898316

[45] Chirumbolo S Ortolani R Veneri D Raffaelli R Peroni D Pigozzi R Lymphocyte phenotypic subsets in umbilical cord blood compared to peripheral blood from related mothers. Cytometry B Clin Cytom (2011) 80:248–53. 10.1002/cyto.b.20588 21692178

[46] Keever CA Marrow B Characterization of cord blood. J Hematother (1993) 206:203–6. 10.1089/scd.1.1993.2.203 7921975

[47] Matsumoto M Baba A Yokota T Nishikawa H Ohkawa Y Kayama H Interleukin-10-producing plasmablasts exert regulatory function in autoimmune inflammation. Immunity (2014) 41:1040–51. 10.1016/j.immuni.2014.10.016 25484301

[48] Burdin N Rousset F Banchereau J. B-cell-derived IL-10: production and function. Methods (1997) 11:98–111. 10.1006/meth.1996.0393 8990095

[49] Weller S Braun MC Tan BK Rosenwald A Cordier C Ellen M Human blood IgM memory B-cells are circulating splenic marginal zone B-cells harboring a prediversified immunoglobulin repertoire. Blood (2013) 104:3647–54. 10.1182/blood-2004-01-0346 PMC2590648 15191950

[50] Cerutti A Cols M Puga I Marginal zone B-cells: virtues of innate-like antibody-producing lymphocytes. Nat Rev Immunol (2013) 13:118–32. 10.1038/nri3383 23348416 PMC3652659

[51] Allman D Pillai S Peripheral B-cell subsets. Curr Opin Immunol (2008) 20:149–57. 10.1016/j.coi.2008.03.014 18434123 PMC2532490

[52] Muzzio DO Ziegler K Ehrhardt J Zygmunt M Jensen F Marginal zone B-cells emerge as critical component of pregnancy wellbeing. Reproduction (2015) 151(1):29–37. 10.1530/REP-15-0274 26493101

[53] Kessel A Haj T Peri R Snir A Melamed D Sabo E Human CD19^+^CD25^high^ B regulatory cells suppress proliferation of CD4+ T-cells and enhance Foxp3 and CTLA-4 expression in T-regulatory cells. Autoimmun Rev (2011) 11(9):670–7. 10.1016/j.autrev.2011.11.018 22155204

[54] Lobo PI Bajwa A Schlegel KH Vengal J Lee SJ Huang L Natural IgM anti-leukocyte autoantibodies attenuate excess inflammation mediated by innate and adaptive immune mechanisms involving Th-17. J Immunol (2012) 188:1675–85. 10.4049/jimmunol.1101762 22262657 PMC3273570

[55] Lobo PI Schlegel KH Spencer CE Okusa MD Chisholm C McHedlishvili N Naturally occurring IgM anti-leukocyte autoantibodies (IgM-ALA) inhibit T-cell activation and chemotaxis. J Immunol (2008) 180:1780–91. 10.4049/jimmunol.180.3.1780 18209075

[56] Yanaba K Bouaziz J-D Matsushita T Tsubata T Tedder TF The development and function of regulatory B-cells expressing IL-10 (B10 cells) requires antigen receptor diversity and TLR signals. J Immunol (2009) 182:7459–72. 10.4049/jimmunol.0900270 19494269 PMC3733128

[57] Matsushita T Horikawa M Iwata Y Tedder TF Regulatory B-cells (B10 cells) and regulatory T-cells have independent roles in controlling experimental autoimmune encephalomyelitis initiation and late-phase immunopathogenesis. J Immunol (2010) 185:2240–52. 10.4049/jimmunol.1001307 20624940 PMC3717968

[58] Sun CM Deriaud E Leclerc C Lo-Man R Upon TLR9 signaling, CD5+ B-cells control the IL-12-dependent Th1-priming capacity of neonatal DCs. Immunity (2005) 22:467–77. 10.1016/j.immuni.2005.02.008 15845451

