# Supplementary figures and images for: Characterization of the Highly Prevalent Regulatory CD24hiCD38hi B-Cell Population in Human Cord Blood
Source: Front Immunol. 2017 Mar 7;8:201. doi: 10.3389/fimmu.2017.00201 (PMC5339297; doi:10.3389/fimmu.2017.00201)

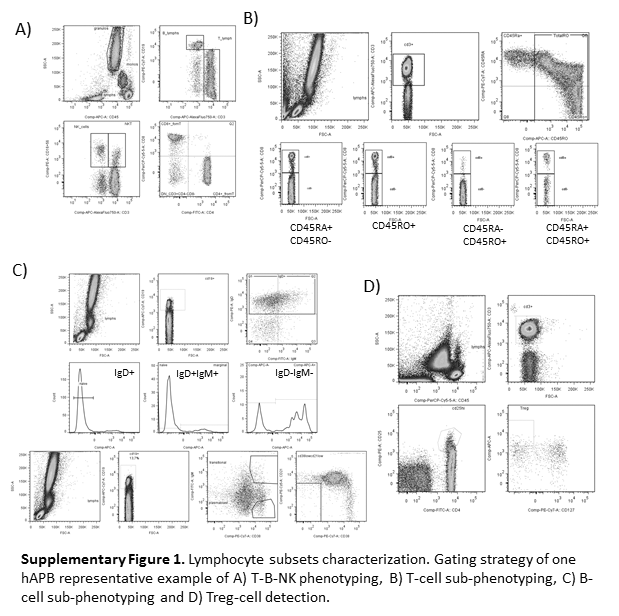

Supplement: Supplementary file 1 [file image_1.tif]

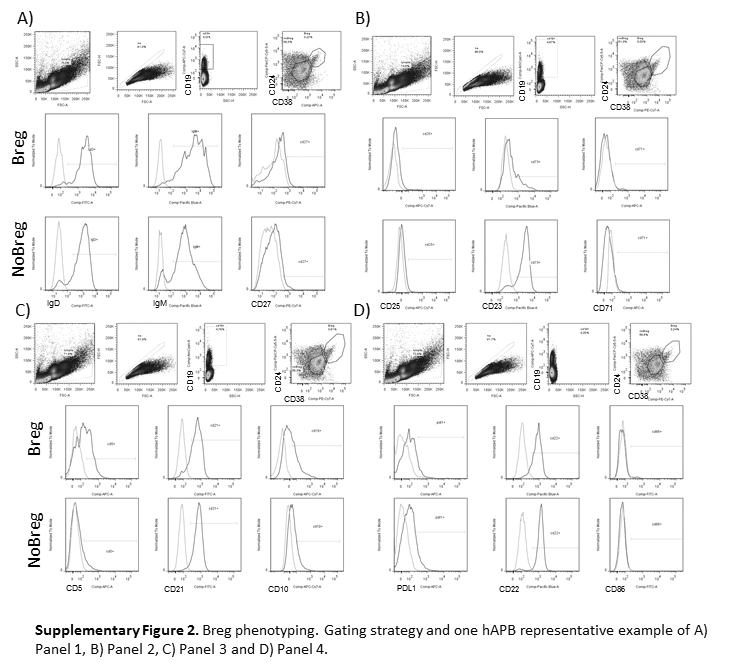

Supplement: Supplementary file 2 [file image_2.tif]

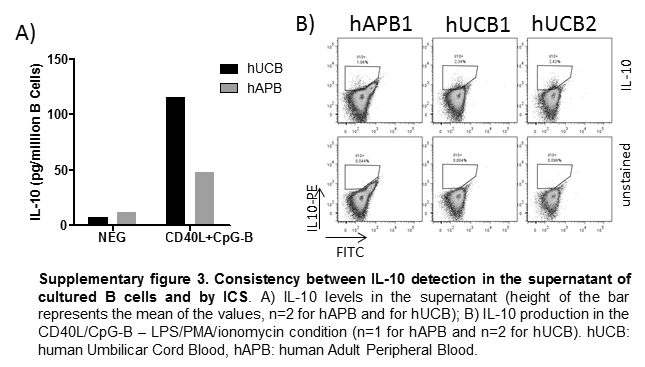

Supplement: Supplementary file 3 [file image_3.tif]
