# Supplementary material for: Characterization of the Highly Prevalent Regulatory CD24hiCD38hi B-Cell Population in Human Cord Blood
Source: Front Immunol. 2017 Mar 7;8:201. doi: 10.3389/fimmu.2017.00201 (PMC5339297; doi:10.3389/fimmu.2017.00201)
Supplement: Supplementary file 4 [file table_1.pdf]

Supplementary Material

Characterization of the highly prevalent regulatory CD24hiCD38hi B cell population in human cord blood

Ana Esteve-Solé[1.2], Irene Teixidó [3], Angela Deyà-Martínez [1.2], Jordi Yagüe [4], Ana M Plaza-Martín [1], Manel Juan [2.4#*], Laia Alsina [1.2#]
# both authors share co-seniorship

*** Correspondence:**
Manel Juan Otero
mjuan@clinic.cat

1 Supplementary Data

Supplementary Material should be uploaded separately on submission. Please include any supplementary data, figures and/or tables.

Supplementary material is not typeset so please ensure that all information is clearly presented. the appropriate caption is included in the file and not in the manuscript, and that the style conforms to the rest of the article.

2 Supplementary Figures and Tables

2.1 Supplementary Tables

Breg. % of	hUCB			hAPB			p
	Mean	±	std	mean	±	std	
Marginal Zone	60.8	±	12.28	4.94	±	1.46	0.1
Naive	30.58	±	7.95	3.26	±	0.65	0.1
IgD^+IgM^+	30.76	±	5.999	3.39	±	0.84	0.057
IgD^+IgM^-	6.016	±	3.625	0.514	±	0.166	0.057
Class Switched	10.8	±	4.363	0.49	±	0.23	0.1

Supplementary Table I. Breg-cell frequency among different B-cell populations
