# Supplementary material for: Characterization of the Highly Prevalent Regulatory CD24hiCD38hi B-Cell Population in Human Cord Blood
Source: Front Immunol. 2017 Mar 7;8:201. doi: 10.3389/fimmu.2017.00201 (PMC5339297; doi:10.3389/fimmu.2017.00201)
Supplement: Supplementary file 5 [file table_2.pdf]

Supplementary Material

Characterization of the highly prevalent regulatory CD24hiCD38hi B cell population in human cord blood

Ana Esteve-Solé[1.2], Irene Teixidó [3], Angela Deyà-Martínez [1.2], Jordi Yagüe [4], Ana M Plaza-Martín [1], Manel Juan [2.4#*], Laia Alsina [1.2#]
# both authors share co-seniorship

*** Correspondence:**
Manel Juan Otero
mjuan@clinic.cat

1 Supplementary Data

Supplementary Material should be uploaded separately on submission. Please include any supplementary data, figures and/or tables.

Supplementary material is not typeset so please ensure that all information is clearly presented. the appropriate caption is included in the file and not in the manuscript, and that the style conforms to the rest of the article.

2 Supplementary Figures and Tables

2.1 Supplementary Tables

	48h CD40L+CpG					
	5h BFA			5h CLIP-B		
	mean	±	std	mean	±	std
**hUCB**	6.06	±	2.08	9.73	±	3.13
**hAPB**	5.63	±	2.54	7.92	±	2.9

	hUCB			hAPB			p
	mean	±	std	mean	±	std	
**Bcell**	3.93	±	1.32	2.287	±	0.97	0.2
**Breg**	5.54	±	2.15	1.92	±	0.63	0.1
**NoBreg**	6.16	±	3.72	1.35	±	0.79	0.1

Supplementary Table II. IL-10 production after maturation ± CLIP-B stimulation (CpG-B/LPS /Ionomycine/PMA + BFA) and spontaneously after 5h incubation in the presence of BFA
