# Supplementary material for: Characterization of the Highly Prevalent Regulatory CD24hiCD38hi B-Cell Population in Human Cord Blood
Source: Front Immunol. 2017 Mar 7;8:201. doi: 10.3389/fimmu.2017.00201 (PMC5339297; doi:10.3389/fimmu.2017.00201)
Supplement: Supplementary file 6 [file table_3.pdf]

Supplementary Material

Characterization of the highly prevalent regulatory CD24hiCD38hi B cell population in human cord blood

Ana Esteve-Solé[1.2], Irene Teixidó [3], Angela Deyà-Martínez [1.2], Jordi Yagüe [4], Ana M Plaza-Martín [1], Manel Juan [2.4#*], Laia Alsina [1.2#]
# both authors share co-seniorship

*** Correspondence:**
Manel Juan Otero
mjuan@clinic.cat

1 Supplementary Data

Supplementary Material should be uploaded separately on submission. Please include any supplementary data, figures and/or tables.

Supplementary material is not typeset so please ensure that all information is clearly presented. the appropriate caption is included in the file and not in the manuscript, and that the style conforms to the rest of the article.

2 Supplementary Figures and Tables

2.1 Supplementary Tables

	T-cell mean ± std	T-cell:Breg mean ± std	Tcell:NoBreg mean ± std	T-cell Breg	T-cell NoBreg	Breg NoBreg
**IFNg**	1.63 ± 0.23	0.93 ± 0.1	1.44 ± 0.21	**0.004**	0.39	0.09
**IL4**	1.66 ± 0.23	0.86 ± 0.16	1.9 ± 0.28	0.02	0.9	0.009

Supplementary table III. Stimulation Ratio of T-cells when co-cultured with Bregs, Cells were co-cultured during 72h in the presence of plate-bound CD3 and stimulated 5h with PMA and Ionomycine in the presence of monensin.
