# Supplementary material for: Characterization of the Highly Prevalent Regulatory CD24hiCD38hi B-Cell Population in Human Cord Blood
Source: Front Immunol. 2017 Mar 7;8:201. doi: 10.3389/fimmu.2017.00201 (PMC5339297; doi:10.3389/fimmu.2017.00201)
Supplement: Supplementary file 7 [file table_4.pdf]

Supplementary Material

Characterization of the highly prevalent regulatory CD24hiCD38hi B cell population in human cord blood

Ana Esteve-Solé[1.2], Irene Teixidó [3], Angela Deyà-Martínez [1.2], Jordi Yagüe [4], Ana M Plaza-Martín [1], Manel Juan [2.4#*], Laia Alsina [1.2#]
# both authors share co-seniorship

*** Correspondence:**
Manel Juan Otero
mjuan@clinic.cat

1 Supplementary Data

Supplementary Material should be uploaded separately on submission. Please include any supplementary data, figures and/or tables.

Supplementary material is not typeset so please ensure that all information is clearly presented. the appropriate caption is included in the file and not in the manuscript, and that the style conforms to the rest of the article.

2 Supplementary Figures and Tables

2.1 Supplementary Tables

		hUCB							hAPB							hUCB vs hAPC	
		Breg			NoBreg			p	Breg			NoBreg			p	Breg	NoBreg
		mean	±	std	mean	±	std		mean	±	std	mean	±	std			
**IgM**	%	98.6	±	0.194	95.99	±	1.23	0.029	94.83	±	2.2	88.58	±	0.45	0.100	0.057	0.057
	MFI	14380	±	1758	4953	±	318.4	0.029	7716	±	1278	2706	±	255.8	0.100	0.057	0.057
**IgD**	%	93.78	±	1.43	95.84	±	0.17	0.340	92.5	±	2.85	90.33	±	0.4	0.700	>0.9999	0.057
	MFI	2918	±	326.8	2297	±	105.5	0.114	3139	±	139.9	2434	±	187	0.100	0.400	0.630
**CD27**	%	0.652	±	0.21	0.9207	±	0.45	>0.9999	6.108	±	1.209	5.338	±	0.53	>0.9999	0.100	0.100
	MFI	263.8	±	35.35	134.3	±	18.34	0.100	301	±	1.26	147	±	5.57	0.100	0.700	0.700
**CD10**	%	35.48	±	6.73	13.66	±	1.49	0.030	32.65	±	0.93	15.51	±	2.07	0.100	>0.9999	0.630
	MFI	15.8	±	15.7	37.16	±	4.06	0.340	25	±	0.95	45.35	±	5.21	0.700	0.620	0.230
**CD5**	%	26.43	±	2.88	26.01	±	3.91	0.690	24.74	±	7.84	6.59	±	1.89	0.200	0.860	0.057
	MFI	223.4	±	20.75	202.5	±	22.49	0.880	179.1	±	22.04	39.88	±	7.88	0.100	0.200	0.057
**CD25**	%	4.373	±	0.416	2.776	±	0.129	0.030	5.822	±	0.85	2.142	±	0.34	0.100	0.220	0.220
	MFI	7.46	±	0.93	54.33	±	11.1	0.030	20.66	±	11.37	276.2	±	182.6	0.100	0.230	0.230
**CD73**	%	13.45	±	2.92	50.83	±	10.79	0.029	37.98	±	8.23	85.7	±	2.49	0.100	0.057	0.057
	MFI	431.4	±	77.81	918	±	226.8	0.200	1185	±	515.2	2623	±	342.7	0.200	0.057	0.057
**CD21**	%	71.43	±	7.31	91.03	±	2.79	0.057	72.3	±	6.6	90.78	±	2.03	0.100	0.860	0.860
	MFI	631.5	±	85.22	770.8	±	87.99	0.490	584.3	±	90.23	624.2	±	71.47	0.700	0.230	0.400
**CD22**	%	90.53	±	4.43	92.96	±	3.69	0.690	89.08	±	0.28	95.17	±	1.39	0.100	0.400	0.860
	MFI	800.9	±	44.77	809.4	±	36.15	>0.9999	1293	±	109.3	1226	±	50.23	>0.9999	0.057	0.057
**CD71**	%	4.058	±	1.06	4.85	±	1.59	0.890	6.955	±	2.804	6.72	±	2.222	>0.9999	0.860	0.630
	MFI	120.3	±	30.34	117.1	±	29.8	>0.9999	63.95	±	18.95	66.32	±	10.14	>0.9999	0.230	0.400
**CD86**	%	0.58	±	0.1	1.02	±	0.23	0.110	5.8	±	3.87	1.22	±	0.59	0.200	0.057	0.630
	MFI	77.28	±	9.84	77.98	±	9.909	>0.9999	69.42	±	8.89	53.22	±	4.73	0.200	0.630	0.230
**PDL1**	%	15.89	±	7.056	15.1	±	6.49	0.890	9.5	±	2.07	13.12	±	1.289	0.400	>0.9999	>0.9999
	MFI	439.4	±	19.41	309.3	±	62.12	0.200	385.8	±	174.8	166.8	±	3.87	0.400	0.630	0.057

Supplementary Table IV. Phenotype markers among hAPB and hUCB Breg and NoBreg cells.
